# The Neural Bases of Graphical Perception: A Novel Instance of Cultural Recycling?

**DOI:** 10.1162/JOCN.a.81

**Published:** 2026-01-01

**Authors:** Lorenzo Ciccione, Stanislas Dehaene

**Affiliations:** 1 Cognitive Neuroimaging Unit, CEA, INSERM, Université Paris-Saclay, NeuroSpin Center, 91191 Gif/Yvette, France; 2 Collège de France, Université Paris Sciences Lettres (PSL), 11 Place Marcelin Berthelot, 75005 Paris, France; 3 Department of Psychology, Université Paris 8, DysCo Lab, 93526 Saint-Denis, France

## Abstract

Graphical representations of quantitative data abound in our culture, and yet the brain mechanisms of graphicacy, by which viewers quickly extract statistical information from a data graphic, are unknown. Here, using scatterplots as stimuli, we tested two hypotheses about the brain areas underlying graphicacy. First, at the perceptual level, we hypothesized that the visual processing of scatterplots and their main trend recycles cortical regions devoted to the perception of the principal axis of objects. Second, at a higher level, we speculated that the math-responsive network active during arithmetic and mathematical truth judgments should also be involved in graphical perception. Using fMRI, we indeed found that the judgment of the trend in a scatterplot recruits a right lateral occipital area involved in detecting the orientation of objects, as well as a right anterior intraparietal region also recruited during mathematical tasks. Both behavior and brain activity were driven by the *t* value that indexes the statistical correlation in the data, and right intraparietal activation covaried with participants*’* graphicacy level. On the basis of this first approach to the neural bases of graphical perception, we suggest that, like literacy and numeracy, graphicacy relies on the recycling of brain areas previously attuned to a similar problem, here the perception of object orientation.

## Introduction

Consider the following three statements: “The stock value of the company I invested in is skyrocketing”; “The number of daily Covid deaths has increased over the past few months”; “In humans, height correlates with weight.” What do these observations have in common? They can all be depicted graphically as scatterplots or curves in a 2-D *x–y* coordinate frame, one of the most widely used methods in data visualization (Friendly & Denis, 2005). The reader accustomed to dealing with graphical representations probably even imagined those sentences in a graphical format while reading. And this would not be surprising, because scatterplots and other data graphics more generally are widespread in many fields ranging from journalism to scientific research and from medicine to economics.

The abundant use of data graphics in the contemporary world is quite recent, however. Data graphics were invented only in the past 3 centuries (Friendly, 2008; Tilling, 1975), much more recently than writing and numerical/mathematical notation, the other two main visual cultural products in human history. Comparably, whereas years of scientific research have begun to elucidate the cognitive and brain processes underlying literacy and numeracy (Nieder, 2019; Rayner, Pollatsek, Ashby, & Clifton, 2012; Dehaene, 2010, 2011), including their developmental trajectory (Dehaene, Cohen, Morais, & Kolinsky, 2015; Cain, 2010; Butterworth, 2005), very little is known about the behavioral and brain mechanisms of “graphicacy,” the ability to perceive and interpret graphics (Balchin & Coleman, 1966). The present work provides a first attempt at elucidating them.

Our research was developed within the general framework of cultural neuronal recycling (Dehaene & Cohen, 2007; Dehaene, 2005). Both literacy and numeracy have been found to rest on evolutionarily ancient cognitive systems initially involved in other functions, yet whose plasticity is sufficient to reorient them toward a novel cultural function: Literacy relies on a preexisting circuit for object and face recognition and its connections to the language system (Agrawal & Dehaene, 2024; Saygin et al., 2016; Dehaene et al., 2015; Hannagan, Amedi, Cohen, Dehaene-Lambertz, & Dehaene, 2015), whereas numeracy preempts regions involved in numerosity perception and the estimation of approximate quantities (Dehaene, 2011; Piazza, 2010). According to the neuronal recycling hypothesis, novel cultural inventions such as the alphabet or Arabic numerals, to be learnable, must capitalize on pre-existing, evolutionary ancient brain circuits that are plastic enough to be partially repurposed. Neuronal recycling may explain how culturally learned and relatively recent skills in human evolutionary history can nevertheless be quickly and intuitively acquired, based on preexisting perceptual and cognitive skills or “core knowledge” (Spelke, 2003), and end up being associated with well-delimited regions of the human brain that are reproducible across individuals and across cultures, such as the visual word form area.

Neuronal recycling is often misunderstood as requiring a radical reorientation of cortical function, including a partial or total replacement of the evolutionary earlier function by the new cultural one. Such radical repurposing may occur, as when the acquisition of literacy competes with face recognition in ventral visual cortex (Dehaene et al., 2015), but it does not have to. For instance, the nearly-universal cultural tradition of creating masks that mimic and distort the human face (Sperber & Hirschfeld, 2004) is a specifically human cultural invention (no other animal species has), yet one that likely relies on the pre-existing circuit for face recognition, including its ability to generalize to distorted or caricatural faces, without requiring it to be strongly repurposed. Here, we propose that the perception of data graphics may be similar: fundamental to their invention is the idea of displaying considerable data in a form that makes it easily graspable by our preexisting visual object perception processes, without necessarily requiring them to abandon their prior function.

At present, little is known about the mechanisms of graphicacy at the behavioral level, and nothing at the neural level. Behavioral studies have focused on improving the design of graphical representations (Franconeri, Padilla, Shah, Zacks, & Hullman, 2021), deciding what makes plots effective (Kim & Heer, 2018), memorable (Peña, Ragan, & Harrison, 2020; Borkin et al., 2013) or attention-grabbing (Borkin et al., 2016). Other research has attempted to characterize the origins of perceptual biases when reading data (Ciccione, Dehaene, & Dehaene, 2023; Xiong, Stokes, Kim, & Franconeri, 2023) and understanding how human participants make subjective or numerical estimates from a graphic data set, such as estimating the mean correlation between variables (Rensink, 2017; Bobko & Karren, 1979) or the barycenter of a scatterplot (Hong, Witt, & Szafir, 2021). More recently, using classical methods from psychophysics, our group has begun to investigate the perceptual mechanisms of scatterplot perception (Ciccione, Dehaene, et al., 2023; Ciccione, Sablé-Meyer, et al., 2023; Ciccione & Dehaene, 2021). Using a trend judgment task, where participants decided whether the data are going up or down, we found that humans, regardless of culture, age, and education, can be quite accurate in this task and base their trend judgment on a statistical summary index of the data, the *t* value of the corresponding regression. Thus, humans perform close to an optimal statistician when it comes to scatterplot perception.

In the current study, we capitalize on this task to perform the first brain-imaging study of the neural bases of graphicacy and more specifically of the human ability to extract intuitive statistical information from graphics. Specifically, we aimed to answer the following four research questions:

1) What are the main brain areas involved in trend judgment? To answer this question, we designed an experimental paradigm using fixed stimuli but different tasks. Participants saw a series of scatterplots composed of triangles and diamonds, and, for each of them, they either had to judge the trend in the graphic (ascending or descending) or, in other blocks, the most numerous shape (triangles or diamonds). The first task is clearly graphical in nature: To judge the trend, the observer has to treat the scatterplot as a whole and analyze it for its dominant orientation. The second task, however, while using identical stimuli, does not require any global graphical treatment, but only a focus on the shapes of the local symbols and an estimate of their proportions. Performing a simple univariate contrast between the two types of blocks should yield a first idea of the brain areas recruited by the trend judgment task, while subtracting away the regions involved in the mere perception of the individual data points.
2) Does the neuronal recycling hypothesis, which has been proposed for literacy and numeracy, also apply to graphicacy? As mentioned above, the hypothesis postulates that culturally learned skills, such as reading and arithmetic, are implemented by a repurposing of brain areas initially devoted to a distinct but related function. In the case of noisy scatterplots, what function might be recycled? Here, we propose that it recycles the fast extraction of the principal axis of objects. The hypothesis is that, at a certain level of abstraction, a scatterplot, although composed of multiple dots, is treated as a single visual object (e.g., an ellipse-like probability distribution). The perceptual stage of graphic processing, where participants decide on the main trend of the data, may therefore recycle the evolutionary old ability to detect the orientation of an object and, more generally, its “skeleton” (i.e., the organization, orientation, size and width of its main axes; Ayzenberg, Chen, Yousif, & Lourenco, 2019; Bodily, Sullens, Price, & Sturz, 2018; McCloskey, Valtonen, & Sherman, 2006).
  An interesting behavioral finding that already lends support to this idea is that, when asked to place an optimal regression line on a scatterplot, humans do not match the slope of the classical ordinary least-squares analysis (ordinary least-squares regression), but perform a Deming regression that corresponds to extracting the principal axis of the scatterplot (Robinson, Howard, & VanderPlas, 2023; Ciccione & Dehaene, 2021; Mosteller, Siegel, Trapido, & Youtz, 1981). This computation is formally identical to the one involved in the extraction of the principal axis of a visual object.
  Our hypothesis predicts that graphic trend judgment and object orientation involve shared cortical mechanisms. To test this prediction, we included in our fMRI study an object orientation task. The experimental paradigm was strictly parallel to that of graphical perception (“same stimuli, different tasks”): Participants viewed simple objects (e.g., a fork) and were asked to judge either their orientation (ascending or descending) or, in other blocks, their category (kitchen tool or not). We once again reasoned that, because both tasks used the same stimuli, any difference in neural activity would reflect the specific cognitive process at play for each task and thus isolate the areas involved in the extraction of object orientation. The neuronal recycling hypothesis predicted that those areas would overlap closely with those involved in trend judgment.
3) Is trend judgment sustained by areas also involved in numerical and mathematical cognition? In an important early theorization of graphical perception, Pinker (1990) proposed that following the perceptual stage, the processing of data graphics involves a higher-level of semantic interpretation where spatial dimensions are interpreted as meaningful quantities and meaningful mathematical relations between the *x* and *y* variables are extracted. Although the present trend judgment task does not emphasize this high-level interpretation of the graph, prior behavioral findings indicate that the precision with which participants perform trend judgments on noisy scatterplots is tightly correlated with their numerical and mathematical knowledge and education (Ciccione, Sablé-Meyer, et al., 2023). Similar correlations have been found with the capacity to extrapolate nonlinear functions such as exponential trends (Ciccione, Sablé-Meyer, & Dehaene, 2022) and other graphic-based tasks (Ludewig, Lambert, Dackermann, Scheiter, & Möller, 2020). Those observations suggest that even a task as simple as trend judgment does not stop at an early perceptual level but also involves a mathematical stage. To investigate whether this hypothesis holds at the brain level, we asked participants to take a classic fMRI localizer (Pinel et al., 2007; Pinel, Piazza, Le Bihan, & Dehaene, 2004), which involves, among other contrasts, the performance of mental arithmetic calculations relative to the comprehension of sentences, both using a similar written input. We then looked at the overlap between areas active during mental arithmetic and during trend judgment.
4) Is brain activation modulated by the *t* value, the statistical index that summarizes the strength of the correlation in a scatterplot? Previous behavioral studies showed that participants*’* accuracy and RTs were well subsumed by the *t* value associated to the graphic (Ciccione, Sablé-Meyer, et al., 2023). We thus wondered whether we could observe such a parametric dependence at the brain level. To this aim, we examined whether and where activation was modulated by the *t* value of the scatterplot. For these fMRI runs, we used an event-related graphical perception paradigm, with trials separated by a long and jittered interval, thus permitting single-trial analysis. On every trial, participants saw a scatterplot that varied in slope (positive or negative), noise level (large or small), number of points (18 or 38), and the visual hemifield in which it appeared (left or right). The hemifield manipulation was introduced to eliminate retinotopic explanations of our findings and investigate the pure effects of the scatterplot parameters independently of the visual area they covered. Concretely, we performed a parametric regression analysis of the effect of the *t* value on brain activity.

**Figure 1:**
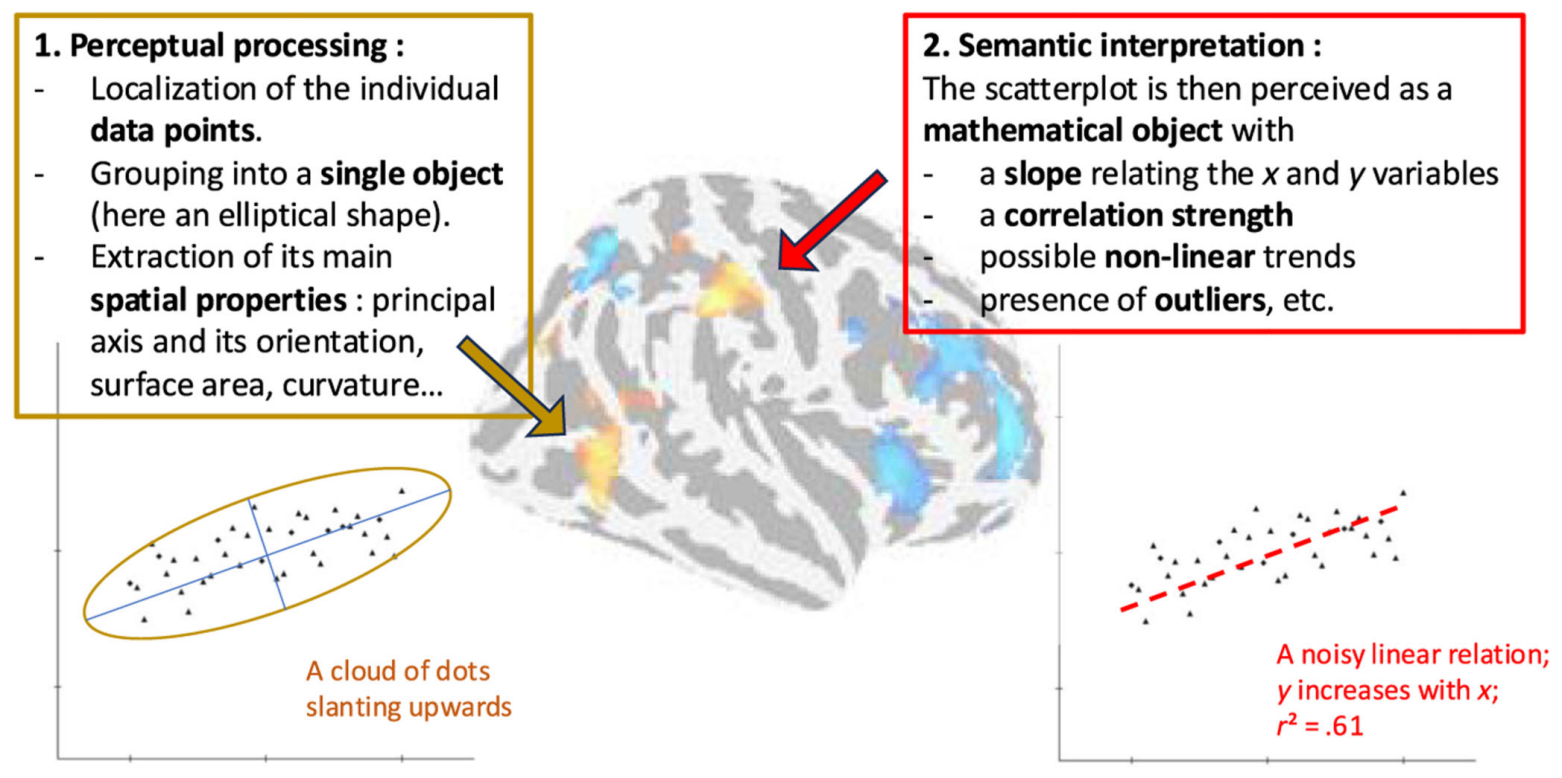
A tentative two-stage model of graphical perception. During the perceptual stage, which is proposed to recycle object perception abilities, the cloud of data points is processed as a single object whose principal axis and its orientation are extracted. Other parameters such as surface area (useful to estimate correlation strength), curvature (an index of nonlinearity), and medial axis (useful to estimate the type of mathematical function) may also be extracted. In a second stage, those perceptual features are used to guide mathematical interpretation: The slope is interpreted as a relation between *x* and *y* variables, whose strength and functional relationship can also be evaluated. The fMRI activity presented in the Results section, shown in the background, suggests that the perceptual stage may rely on the right posterior inferior temporal gyrus, and the second stage on the right aIPS.

Our predictions are summarized in the tentative model presented in Figure 1. The model supposes that graphical perception follows two consecutive stages. During the perceptual stage, which recycles object perception abilities, the cloud of dots is grouped and its principal axes are extracted. This oriented object is then passed on to the second stage of semantic interpretation, during which it is interpreted in mathematical terms. In particular, the graphic is interpreted as a relationship between the *x* and *y* variables, and its slope, linearity, strength, and functional form are encoded. Whereas the first stage is thought to be universal and shared with nonhuman primates (Ciccione, Dighiero-Brecht, Claidière, Fagot, & Dehaene, 2024), although it may still vary with perceptual experience, the second stage, being semantic in nature, should depend on the participants*’* graphicacy level, which itself correlates with mathematical competence and education (Ciccione, Sablé-Meyer, et al., 2023). We used fMRI to provide an initial test of those ideas.

## Methods

### Ethics

The study was conducted in agreement with the Declaration of Helsinki and French bioethics laws. It was also approved by the French ethics board for biomedical research (CPP 100050).

### Participants

The experiment was advertised on the laboratory recruitment platform. Participants had to meet the following criteria to be considered eligible: being French native speakers, being between 18 and 35 years old, not taking psychoactive drugs, not being pregnant, having normal or corrected-to-normal vision, being able to move their hands and fingers, not having metal implants in the body, and being a university student in STEM or having completed at least 3 years of university studies in STEM subjects. The latter criteria aimed at ensuring a relative homogeneity of the sample in terms of their mathematical and general knowledge and they allowed to make sure that, for all participants, graphics were meaningful cultural objects. Twenty adult participants were recruited (12 women, 8 men; mean age: 25.2 ± 4.1). The sample size was determined by rounding to the nearest 10 the median sample size of neuroimaging articles published in top neuroimaging journals in 2017 (Szucs & Ioannidis, 2020). Half of participants performed the experiment starting with the response-hand configurations described below, whereas the other half started with the opposite order.

### Procedure

**Figure 2:**
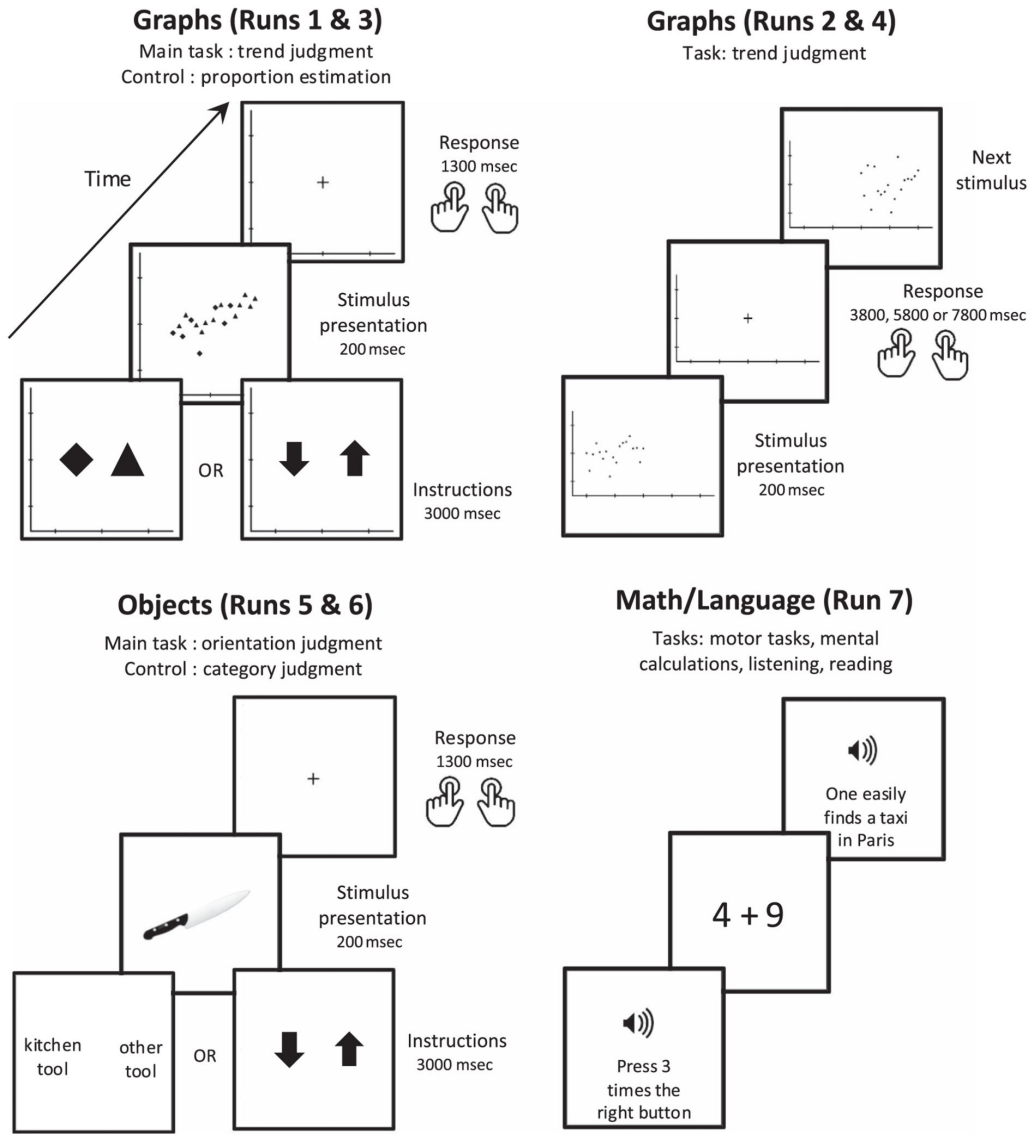
Organization of the experiment. Runs 1 and 3, using a block design, aimed to localize the areas activated during graphic trend judgment relative to a control proportion estimation task using the same stimuli (noisy scatterplots made of equiluminant diamonds and triangles). The two tasks alternated in miniblocks of eight trials each. Runs 2 and 4, using a single-trial design, examined the variations in activation during the trend judgment task. The same stimuli were used, except that the graphics randomly appeared in the left or in the right hemifield. Runs 5 and 6, using a block design, aimed to localize the areas activated during orientation judgment relative to categorization of the same stimuli (oriented objects). Again, the two tasks alternated in miniblocks of eight trials each. Run 7 consisted of a previously published localizer for a series of arithmetic, linguistic (reading/ listening), and motor tasks (Pinel et al., 2007).

The experimental session was divided into seven runs (see Figure 2), plus an anatomical T1 scan. Before entering the scanner, each participant was instructed on how to perform each run and took a few practice trials. Practice was intended uniquely to check for the correct execution of the task (e.g., giving only a single response per trial, fixating the center of the screen, not moving the head).

#### Run 1 and Run 3: Trend Judgment versus Proportion Estimation (Blocked Paradigm)

In those runs, the stimuli were noisy scatterplots consisting in a variable proportion of triangles and diamonds. At the beginning of each miniblock of eight trials, participants saw one of two possible instructions for 3000 msec. In the control condition, before the first type of miniblock, a diamond and a triangle appeared, telling participants to concentrate, for the following eight trials on a proportion estimation task where they were asked to decide which shape was the most numerous. The order of shapes specified the response assignment: For instance, if the display showed a diamond left of a triangle, they had to press the left button if they thought there were more diamonds, and the right button if they thought there were more triangles. In the graphicacy condition, before the other type of miniblock, an upward and a downward arrow appeared, instructing participants that they had to judge the trend of the scatterplot (descending or ascending). Again, the order of the arrows on screen indicated that, for instance, they were asked to press the right button for ascending stimuli and the left button for descending ones.

Note that, by design, both tasks concerned mathematical objects (graphical perception vs. estimation of a proportion of geometrical shapes). We selected on purpose a control task that was not just perceptually but also cognitively related to the main task, so that any difference in neural activity for the trend judgment could reasonably be attributed uniquely to that specific cognitive process, all other things being equal both in terms of low-level visual processing and in terms of any generic involvement of mathematical or proportional thinking. In addition, pilot work indicated that the proportion estimation task was slightly harder than the trend judgment task (see the Results section), so that the difference in activation for the latter could not simply be attributed to a larger involvement of attentional resources (for a similar approach, see, e.g., Kanwisher, McDermott, & Chun, 1997).

In both cases, the graphic stimulus remained on screen for 200 msec and then a fixation cross appeared for 1300 msec, signaling the response window. Participants were asked to give one answer within that time frame. After eight trials, the fixation cross remained on screen for 3800, 5800, or 7800 msec (on average for 5800 msec) and then a new block of trials started with the relevant instructions showing up, again, for 3000 msec. Run 1 and Run 3 both comprised 20 miniblocks each (10 for proportion detection and 10 for trend judgment). Within each run, miniblock order was randomly determined and not known in advance by participants. The only difference between Run 1 and Run 3 was in the response-hand configuration: Run 3 had the opposite configuration of triangle/diamond and up-arrow/down-arrow as Run 1. Each run lasted 420 sec.

The scatterplot stimuli were created as in our previous studies (Ciccione & Dehaene, 2021). Each plot comprised two unlabeled lines denoting the *x* and *y* axes, each marked with three small ticks at the values 0, 0.5, and 1. The generative program worked as follows. First, the desired number of dots (referred to as *n*) was used to determine the *x* coordinates, which were evenly spaced within the range of 0*–*1. Next, the *y* coordinates were determined according to equation *y*_*i*_ = *αx*_*i*_ + ε_*i*_, where *α* denotes the desired slope and ε_*i*_ are random numbers drawn independently from a normal distribution centered on zero, with a standard deviation σ. In cases where a point occasionally obtained an unusually high or low *y* coordinate (*y* < −0.27 or *y* > 1.27), thus exceeding the *y* axis boundaries, the algorithm restarted for that specific trial. New stimuli were generated independently for each trial and participant. Lastly, *y* coordinates were vertically adjusted by subtracting the mean, ensuring that the underlying regression line passed through the precise center of the screen (i.e., the point P with coordinates *x* = 0.5 and *y* = 0.5). Each data point was represented by either a white diamond or a white triangle, both having the same luminance on screen (i.e., the same number of pixels).

**Figure 3:**
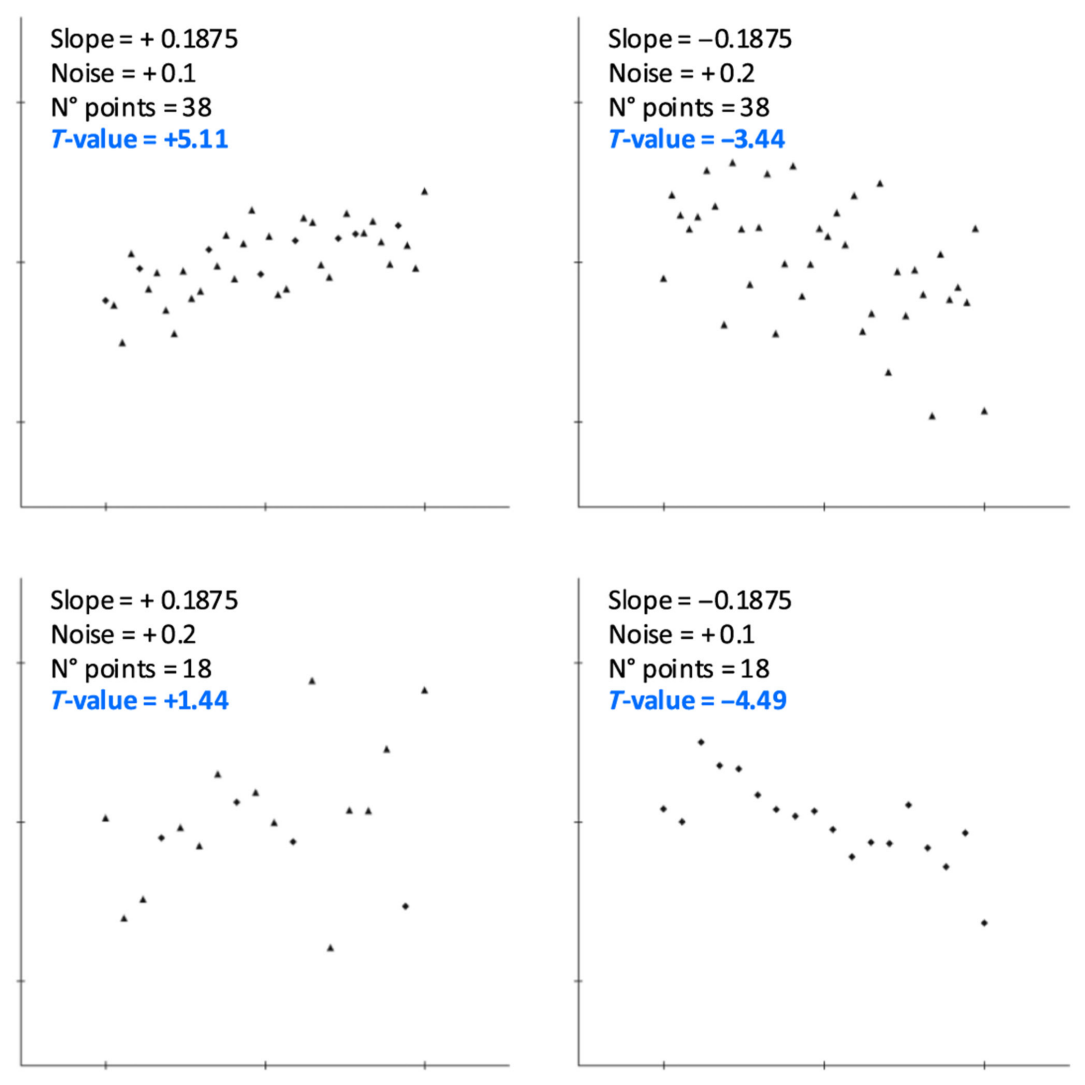
Examples of stimuli. Four different stimuli (with the related generative parameters) are presented.

The experimental factors were the slope of the generative regression (*α* = +0.1875 or −0.1875), the number of items (*n* = 18 or 38), and the noise level (σ = 0.1 or 0.2). Their combination resulted in eight experimental conditions, and one scatterplot per condition was presented in each miniblock, in a random order, for four ascending trends and four descending trends (participants were unaware of these distributions). Of those eight scatterplots, each was assigned one of the following eight ratios of diamonds and triangles (all ratios were presented in a given block): 0 diamonds (i.e., only triangles); 2 diamonds out of 18 or 4 out of 38; 4/18 or 8/38; 6/18 or 12/38; 12/18 or 26/38; 14/18 or 30/38; 16/18 or 34/38; and all diamonds (i.e., 0 triangles). As a result, each miniblock comprised four scatterplots with a majority of triangles and four with a majority of diamonds; again, participants were unaware of these distributions. One hundred sixty trials was presented in each run. Each stimulus subtended a visual angle of 15°. Examples of stimuli are provided in Figure 3.

#### Run 2 and Run 4: Trend Judgment (Event-related Paradigm)

These two runs consisted solely in a trend judgment task, with stimuli presented at a much slower pace allowing for single-trial analysis. Therefore, no mixture of triangles and diamonds was presented in the scatterplots, but only classic circular dots. Scatterplots were again generated according to the same algorithm described above. The experimental factors were the same as above, plus the hemifield in which the scatterplot was presented (left or right). Their combination resulted in 16 experimental conditions, each of which was presented in a random order 8 times during the run, for 128 stimuli per run. Each stimulus had a visual angle of 15°.

The response-hand configuration of Run 2 was the same of Run 1, and the configuration for Run 4 was the same of Run 3. To allow for a single-event design, the intertrial interval was considerably slowed down and jittered, with the fixation cross presented for 3800, 5800, or 7800 msec (on average, 5800 msec). Each run lasted 774 sec.

#### Run 5 and Run 6: Object Categorization and Object Orientation Detection (Blocked Paradigm)

These two runs were structured identically to Run 1 and Run 3, but each trial consisted in the presentation of oriented objects instead of scatterplots. Six objects with a clear principal axis could appear: a knife, a fork, a spoon, a pen, a wrench, or a brush. Each was rotated to six possible orientations: −45°, −30°, -;15°, +15°, +30°, and +45°, thus defining 36 possible stimuli. Two tasks were proposed to participants. On half of the miniblocks, they were asked to categorize the object as belonging to the kitchen or not. This task was cued by the letters “C” and “A,” C standing for “cuisine” (*kitchen* in French) and A standing for “autre” (*other* in French). On the other miniblocks, they had to judge the orientation of the object (cued by the same up and down arrows as in the trend judgment task). Run 6 had the opposite response-hand configuration. Each block comprised four kitchen tools and four other types of tools and, independently, four upward and four downward objects (again, participants were unaware of these distributions). All objects were oriented with their graspable portion on the left. All other parameters were identical to Runs 1 and 3. Note that the object Runs were proposed at the end of the experimental session (Runs 5 and 6) to avoid priming participants to conceive the graphics as objects when performing the trend judgment. Note also that, for both Runs 1 and 3 and Runs 5 and 6, instruction screens were displayed only at the beginning of each block and not during the actual task performance. Therefore, and although identical screens were used for the object orientation and graphical perception tasks, such instructions screens could not have directly contributed to the brain activations on a trial-by-trial basis.

#### Run 7: Localizer of Numerical Cognition and Language Networks

The last run consisted in a series of language and mathematical tasks aimed at defining, for each participant, their math-responsive and language-processing networks. The precise procedure for this classic localizer run has been described in detail elsewhere (Pinel et al., 2004, 2007).

### MRI Acquisition Parameters

MRI scans were acquired on a 3 T scanner (Siemens, Tim Trio) equipped with a 64-channel head coil. All functional scans used a T2*-weighted gradient EPI sequence (69 interleaved slices, repetition time = 1.81 sec, echo time = 30.4 msec, voxel size =2×2×2 mm, multiband factor = 3, flip angle = 71°, posterior to anterior phase encoding direction). For accurate anatomical reconstruction, we also obtained a 3-D T1-weighted structural image, always placed between fMRI Runs 4 and 5 (repetition time = 2.30 sec, echo time = 2.98 msec, voxel size = 1 × 1 × 1 mm, flip angle = 9°). Two spin-echo field maps with opposite phase encoding directions were acquired to estimate distortions: one volume in the anterior-to-posterior direction and one volume in the posterior-to-anterior direction. For the analysis, data were smoothed at 4.0 for the first level models, and at 8.0 for the group analysis.

### fMRI Data Preprocessing and Analysis

Preprocessing was performed using fMRIPREP Version 20.2.5 (Esteban et al., 2019), a tool based on Nipype. For the blocked runs (Runs 1, 3, 5, 6, and 7), we computed first-level models per each subject, with separate regressors for the two tasks (e.g., orientation judgment or categorization). We then conducted group-level univariate contrasts between the regressors of interest. For the event-related runs (Runs 2 and 4), we computed two types of first-level models per each subject, either with discrete stimulus characteristics as separate regressors (e.g., large noise or small noise) or with the standardized *t* value (a continuous factor) as modulator. Group-level contrasts were again performed to test either the difference between regressors of interest (e.g., large noise vs. small noise) or the effect of the modulator variable (i.e., the standardized *t* value). All analyses and figures were realized using Nilearn 0.10.0, a neuroimaging package based on scikit-learn (Abraham et al., 2014). In all cases, the statistical threshold was set at *p* < .001 voxel-wise, with false discovery rate (FDR) correction at *α* < .05.

## Results

### Behavioral Results in Trend Judgment

**Figure 4:**
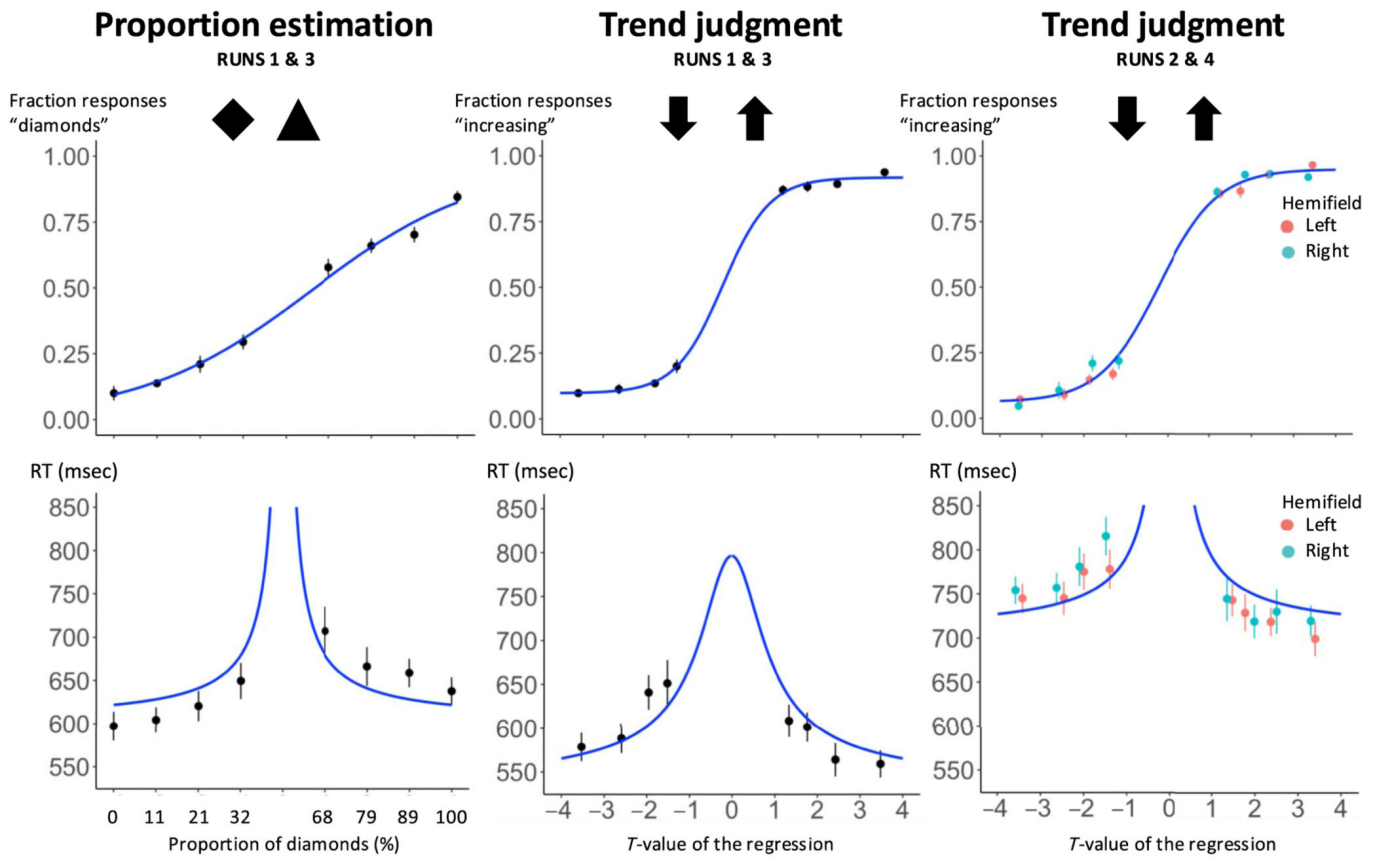
Behavioral performance in the proportion estimation and trend judgment tasks. Top columns show classification rates fitted with a sigmoid, and bottom columns show RTs in milliseconds. The blue line in bottom columns show the prediction of a simple accumulation of evidence model (Gold & Shadlen, 2002).

Behavior was similar in the trend judgment and the proportion estimation tasks (Figure 4). We computed the percentage of responses “diamonds” (for shapes) and the percentage of responses “increasing” (for trends) as a function, respectively, of the proportion of diamonds on screen and of the *t* value of the data set. We found that proportion estimation was slightly harder than estimating the trend of a plot, as indicated by the shallower psychometric function for this task (top row, left plot). Indeed, error rate was significantly higher (24% vs. 14%, paired *t* test, *t*(19) = 7.36, *p* < .001), and RT was slower (640 msec vs. 601 msec, paired *t* test, *t*(19) = 3.11, *p* < .01). In both tasks, RTs were predicted by a simple accumulation of evidence model (Gold & Shadlen, 2002), as indicated by the blue line in bottom columns of Figure 4 (although that basic model did not predict the asymmetries in the data). Note that, because we were mainly interested in the brain activations elicited by the trend judgment task, the discrepancy in difficulty between the two tasks was intended and provides an overcontrol for task difficulty: It means that any larger activation for graphics than for shapes could not be attributed to a higher difficulty or effort devoted to trend judgment, because this task was easier than its control task.

### fMRI Activations during Trend Judgment

**Figure 5:**
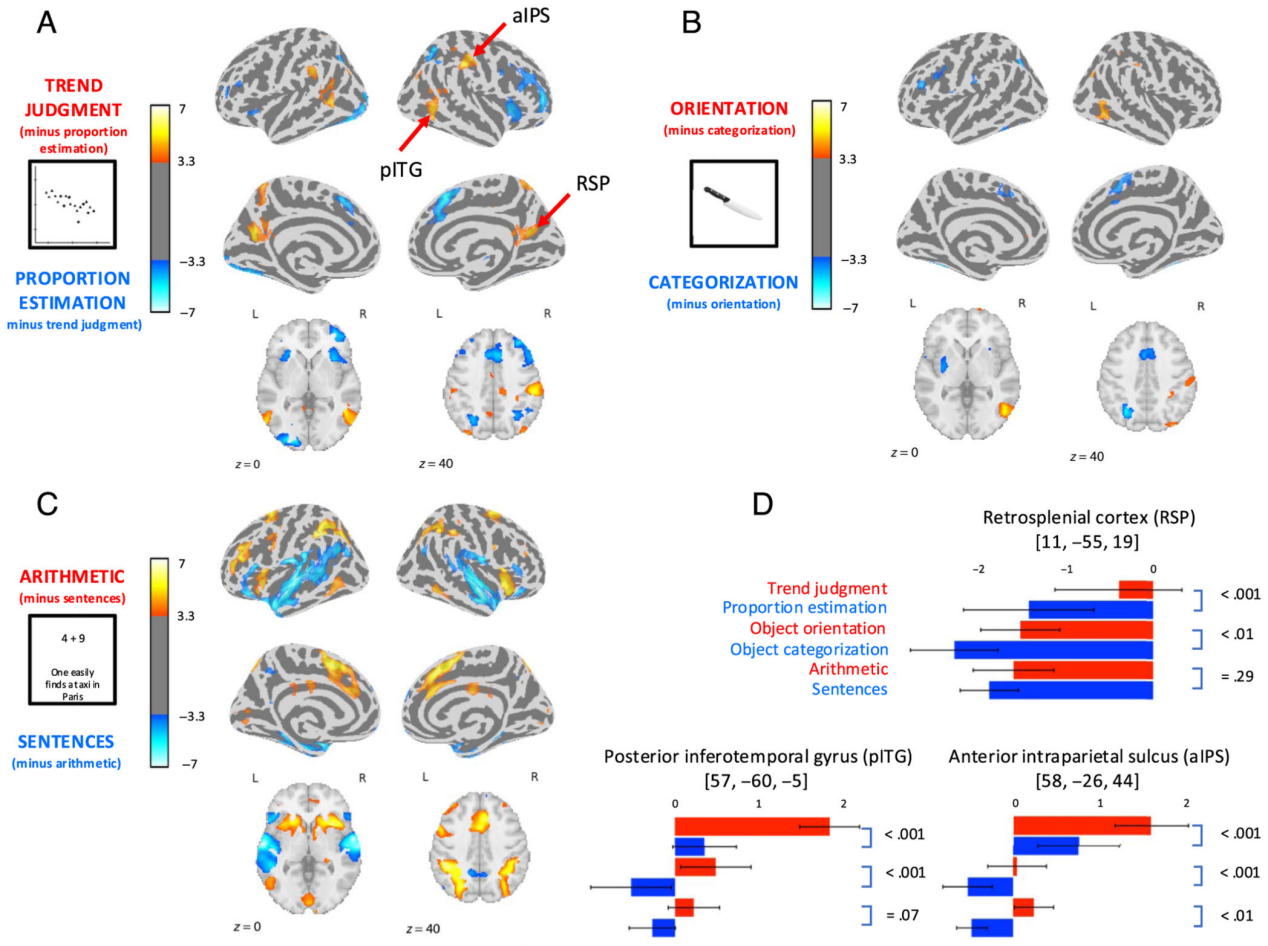
Localization of brain regions activated by graphic trend judgment (A), object orientation judgment (B), and mental arithmetic (C). Each column shows *t* maps for a given task relative to its control task (voxel-wise *p* < .001, FDR α < .05 control for multiple comparisons). Plots in D show the activation levels (beta values, arbitrary units) for each experimental condition at three specific cortical sites, indicated by red arrows in Plot A. Red refers to the main tasks of interest (trend, orientation, and arithmetic), whereas blue refers to control tasks.

We then looked at fMRI activations in the graphics*’* runs (Blocks 1 and 3; see Figure 5A, Supplementary Figure S1, and Supplementary Figure S4). During trend judgment, relative to the nongraphical proportion estimation task, the main activations were in the left posterior superior temporal gyrus, right anterior intraparietal sulcus (aIPS), bilateral retrosplenial cortex, and right posterior inferior temporal gyrus (pITG; group-level analysis, *p* < .001, FDR corrected at *α* < .05; see Supplementary Figure S4 for Montreal Neurological Institute coordinates and a complete list). On the contrary, the proportion detection task (blue areas) significantly activated posterior areas of occipital cortex and large portions of pFC, particularly in the right hemisphere. The latter activation is not surprising, given the higher difficulty of the proportion estimation task compared with trend judgment. Because of this, however, some of the areas with greater activation during trend judgment might actually correspond to a difference in deactivation, with a greater deactivation during proportion estimation. We checked this by masking the *trend judgment > proportion estimation* contrast with the *trend-judgment > rest baseline* contrast. Only the right pITG and aIPS survived, whereas retrosplenial cortex did not. Plots of betas (Figure 5D) confirmed that, in the latter area, there was a deactivation during proportion estimation, but not during trend judgment.

### Relation of Trend Judgment to Object Orientation Judgment

We then analyzed the object runs conducted at the end of the experimental session (Blocks 5 and 6). Behaviorally, the tasks were well matched: The average error rate for orientation judgment (3.9%) was not significantly different from the error rate (4.2%) for categorization (paired *t* test, *t*(19) = −0.51, *p* = .62). Concerning the RTs, categorization was slightly slower than orientation judgments (mean RTs: 544 msec vs. 478 msec; paired *t* test, *t*(19) = 8.9, *p* < .001), again overcontrolling for task difficulty.

At the neural level (Figure 5B, Supplementary Figure S1, and Supplementary Figure S5), the object orientation task yielded greater activation than the control object categorization task primarily in the right pITG, a portion of the right intraparietal sulcus (IPS), and a small area of the right inferior parietal lobule. These activations were all right lateralized. On the contrary, concentrating on object identity significantly activated the left inferior frontal gyrus and other scattered sites (see Figure 5 and Supplementary Figure S5).

Crucial to our goal, the areas active during object orientation judgment largely overlapped with the main region activated for detecting the trend of noisy scatterplots: In the right pITG, the activations to both tasks were virtually identical (Supplementary Figure S1, slice at *z* = 0). This overlap was confirmed at the group level by examining the effect size (beta value) of each experimental condition. When we selected the pITG coordinate where the contrast for object orientation versus object identity was the largest [57, −60, −5], we found that the effect size across subjects was significantly larger for trend judgment than for proportion estimation, *t*(19) = 4.74, *p* < .001 (Figure 5D and Supplementary Figure S4), and the opposite was also true: The pITG coordinate with the highest difference in effect size for trend judgment versus proportion estimation [57, −59, −5] had also a significantly larger effect size for object orientation versus object identity, *t*(19) = 4.95, *p* < .001. We also replicated this finding using subject-specific voxels. To this aim, we defined a 10-mm-radius spherical ROI around the peak coordinate of [57, −60, −5] and, for each subject, within this ROI, identified the 10% voxels most activated in the object orientation > object identity contrast. We then extracted the betas in those same voxels during the graphic task and found a significantly greater activation for trend judgment than for proportion estimation, *t*(19) = 9.59, *p* < .001. The difference between math and linguistic statements was also significant, *t*(19) = 2.91, *p* < .01.

### Relation of Trend Judgment to Arithmetic Processing

We then looked at neural activations elicited when performing mathematical and linguistic tasks. At the group level (Figure 5C and Supplementary Figure S1), the bilateral IPS was significantly more activated by performing arithmetic calculations than by reading or listening to sentences, replicating previous results (Pinel et al., 2004, 2007). Among other areas, the insula, the pre-SMA, and the inferior frontal sulcus were also significantly more activated during math processing compared with linguistic tasks. The full set of clusters significantly activated for math processing is available in Supplementary Figure S3 and Supplementary Figure S6.

Crucially, this network overlapped partially with the areas involved in trend judgment. As clearly visible by comparing Figure 5A and C, math-related activity in the IPS was more posterior than the trend judgment network, which, in turn, seems to stretch till the postcentral gyrus and the inferior parietal lobule. Nevertheless, the two overlapped in the anterior IPS, as confirmed by looking at the effect size (beta value) of each experimental condition in the IPS coordinate where the contrast for trend judgment versus proportion estimation was the largest. At this peak, the activation was significantly larger for math than for language processing as well, *t*(19) = 3.61, *p* < .01 (Figure 5D). However, using the same subject-specific approach as above, although the 10% most active voxels for the contrast trend judgment > proportion estimation were also more active for math processing relative to linguistic tasks, the difference fell short of significance, *t*(19) = 1.87, *p* = .08.

Ventrally, part of the pITG was also more activated for math processing, but this math-related activation extended more anteriorly into the inferior temporal gyrus than the sites previously observed for trend and orientation judgments. As a consequence, the overlap with trend judgment was only partial, and the math > language contrast did not quite reach significance at the peak of the pITG trend-judgment-related activation (*p* = .07; see Figure 5D). At this site, however, the subject-specific approach described above yielded a significant math > language contrast, *t*(19) = 2.67, *p* = .015.

Lastly, we examined whether interindividual differences in graphicacy were reflected in brain activity. At the whole-brain level, no significant effects were found. However, we also performed two subject-specific ROI analyses. Starting with those same 10% most activated voxels in both the aIPS and the pITG, we computed the average raw signal (in arbitrary units) for each subject and correlated such signal with the behavioral performance in the trend judgment task, specifically their “graphicacy index,” which was previously shown to be a proxy for participants*’* intuitive skills in graphical perception (Ciccione, Sablé-Meyer, et al., 2023). We found a significant positive correlation in the aIPS, *t*(18) = 2.21, *r* = .46, *p* < .05, but not in the pITG, *t*(18) = .19, *r* = .04, p = .85.

### Modulators of Trend Judgment Difficulty

Behaviorally, the trend judgment task also yielded information about the determinants of trend judgment responses (Figure 4, right). An ANOVA on trend judgment responses from Runs 1 and 3 showed that the probability to respond “increasing” depended on the orientation of the slope, *F*(1, 19) = 2241.2, *p* < .001, and on its interaction with both the noise level, *F*(1, 19) = 16, *p* < .001, and the number of points in the data set, *F*(1, 19) = 7.8, *p* < .05. As in our previous work, those effects were subsumed by a major influence of the *t* value (assessing the significance of the linear trend in the stimulus graphic) on response choice in a logistic regression analysis of single-trial responses (β = 1.04, *p* < .001). In terms of RTs, participants were significantly affected by the slope, *F*(1, 19) = 18.1, *p* < .001, and the level of noise, *F*(1, 19) = 27.8, *p* < .001, but the number of points did not reach significance, *F*(1, 19) = 1.6, *p* = .22. Again, RT was strongly affected by the absolute value of the *t* value (β = −31.6, *p* < .001; Figure 4, right).

In Runs 2 and 4, scatterplots could appear either on the left visual hemifield or on the right visual hemifield. In a multiple logistic regression on given responses as a function of the graphic *t* value and its hemifield of appearance, we found that participants*’* performance was explained by the *t* value (β = 1.01, *p* < .001) but, crucially, not by the hemifield in which the stimulus appeared (β = .11, *p* = .76), nor their interaction (β = −.05, *p* = .79). The independence of trend judgments from stimulus location thus extends previous behavioral results and suggests that the judgment arises from an abstract, location-independent level of processing.

### Modulators of Brain Activation during Trend Judgment

The event-related design of Runs 2 and 4 allowed to look at univariate effects of the scatterplot features. Supplementary Figure S2 (first row) shows the brain areas significantly more activated for data sets with larger noise than for those with smaller noise (first row). As expected, given that a larger noise level makes the trend perception task more difficult, most of the graphic-related brain network (previously isolated by trend judgment > proportion estimation) showed a greater activation at larger noise levels. Furthermore, noise also increased the activation bilaterally in a dorsal posterior parietal and FEF network, likely related to greater attention and task difficulty. The contrast between graphics with 38 versus 18 data points did not yield any significant result, probably because this variable had a more modest, subthreshold impact on trend judgment difficulty.

**Figure 6:**
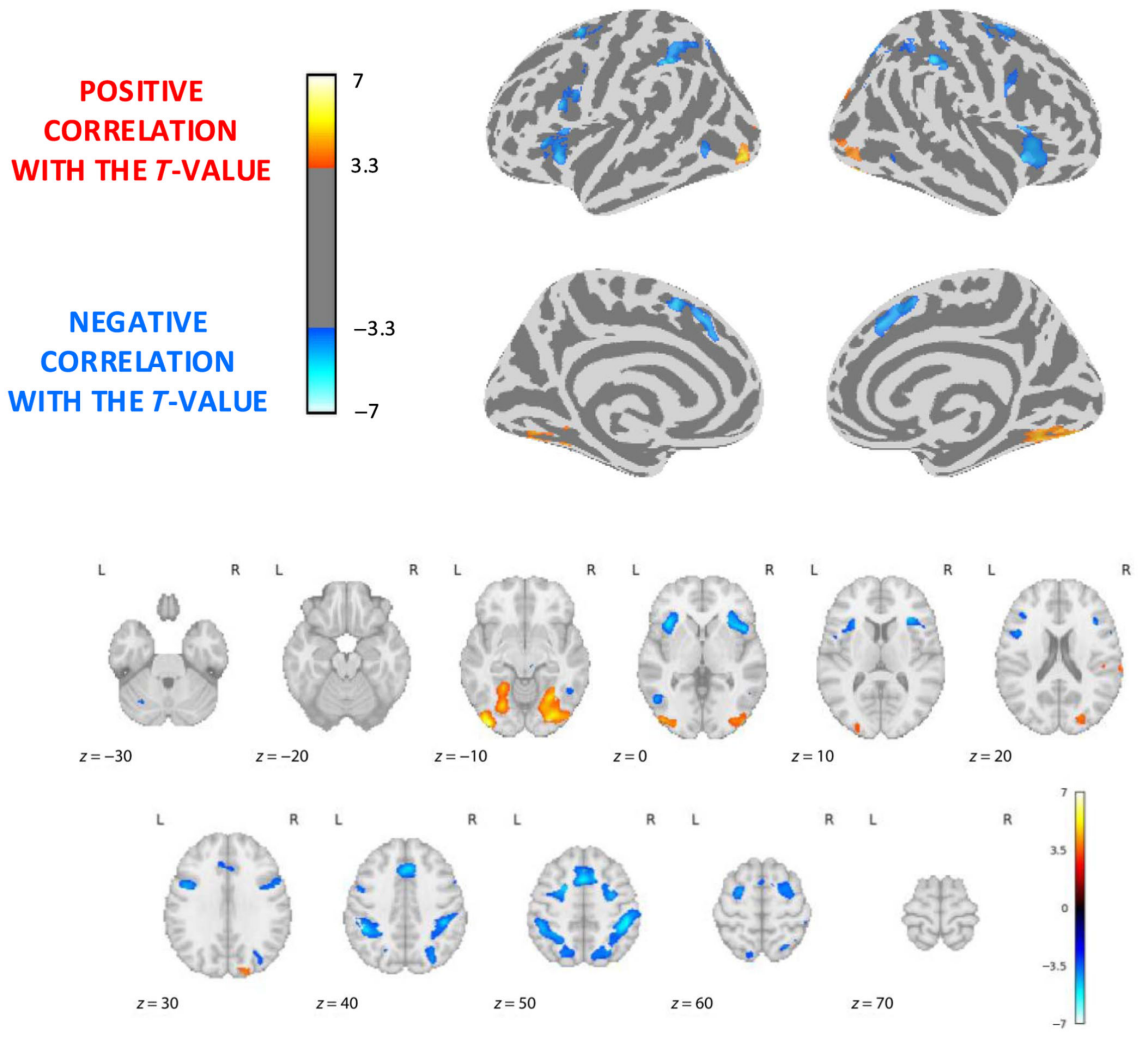
Brain areas modulated by the difficulty of graphic trend judgment. Top: whole-brain view of areas whose activation is positively (red) or negatively (blue) correlated with the *t* value, after regressing out scatterplots*’* noise level. Note that, for greater interpretability, the image was masked to only show areas with a genuine activation (*t* test > 0 in the main comparison of graphical perception relative to baseline). Thus, red/yellow shows regions that are increasingly activated as the graphic correlation increases (making the task easier), and blue shows regions that are increasingly activated as the correlation decreases (making the task more difficult). Bottom: axial cuts through the activations.

Because behavior suggested that the difficulty of the trend judgment task was summarized by the *t* value of the scatterplot (see also Ciccione, Sablé-Meyer, et al., 2023; Ciccione & Dehaene, 2021), we also modeled brain responses in Runs 2 and 4 using the *t* value of each scatterplot (a continuous factor) as a modulator of event-related fMRI activity. Given the above effect of noise level, we also added this variable as an additional control modulator. In this analysis, we considered only areas that were positively activated by the presence of the stimulus, to avoid looking at differences in deactivations. The results appear in Figure 6 (*p* < .001, FDR corrected at *α* < .05). Focusing on negative correlation (given that a lower *t* value makes the trend judgment task more difficult), we indeed found that a large frontal, parietal, anterior cingulate, and anterior insula network increased in activation as the *t* value decreased, even when noise level was controlled for. This network included the right anterior intraparietal site previously associated with trend judgment but, surprisingly, not the right pITG associated with trend and orientation judgments. In the converse direction, a larger *t* value led to increased bilateral activity in ventral occipito-temporal cortex (mostly lingual gyrus, but again not pITG), perhaps indicating that those ventral regions were increasingly responsive when the linear shape of the graphic became increasingly obvious.

Finally, we examined the effects of two peripheral stimulus parameters, namely, graphic orientation and graphic localization. When comparing upward trends versus downward trends, only two small activations were seen in bilateral occipito-parietal cortex (Supplementary Figure S2, second row). On the other hand, the effect of the hemifield of stimulus presentation was largely confined to posterior occipital and left fusiform areas (Supplementary Figure S3). Thus, as expected, all areas previously identified as related to trend judgment were not affected by those lower-level variables.

## Discussion

With a series of psychophysical experiments performed during fMRI at 3 Tesla, we aimed to shed light on the neural bases of graphical perception. In this discussion, we return to the research questions outlined in the Introduction section.

### Brain Areas Involved in Trend Judgment

The first question we asked, given the complete dearth of brain-imaging studies of graphicacy, is what brain areas are active during the perception of scatterplots. We found that trend judgments over noisy scatterplots, relative to a control proportion estimation task on the same stimuli, elicited brain activity mainly in two right-hemispheric inferior temporal and anterior intraparietal regions (Figure 5A), together with an additional recruitment of left pITG and retrosplenial cortex. This network, including its right-hemispheric lateralization, is remarkably consistent with previous studies of the neural coding of the global spatial organization of object parts, relative to their local features (Ayzenberg & Behrmann, 2022; Ayzenberg, Kamps, Dilks, & Lourenco, 2022). More specifically, in agreement with our second goal, here we found a similar activation of the right pITG*—*at its most posterior end abutting lateral occipital cortex (LOC)*—*during graphic trend judgment (as opposed to proportion estimation) and when the same participants judged the orientation of everyday objects (as compared with categorizing the same objects). Previous studies have shown that the LOC, at a site overlapping with the one we observed, is involved in the extraction of the outline of objects and the spatial relations between their subcomponents (Margalit, Biederman, Tjan, & Shah, 2017; Hayworth, Lescroart, & Biederman, 2011), the spatial configuration of figures (Appelbaum, Ales, Cottereau, & Norcia, 2010), the orientation of objects (Hatfield, McCloskey, & Park, 2016), the global shape of objects rather than their local features (Kourtzi & Kanwisher, 2001), and the symmetry of dot configurations around a vertical axis (Cattaneo, Bona, & Silvanto, 2017). The LOC seems to be primarily involved in the detection of overall shape configuration rather than object identity (Kim, Biederman, Lescroart, & Hayworth, 2009) and a nearby region (the lateral occipito-parietal junction) seems specifically involved in detecting the orientation of graspable objects as compared with nongraspable ones (Rice, Valyear, Goodale, Milner, & Culham, 2007). Here, for lack of time within the fMRI, we did not include a contrast for intact versus scrambled objects, which is a classical localizer for LOC (Grill-Spector, Kushnir, Edelman, Itzchak, & Malach, 1998), but future work should determine the precise relation of the present pITG site to the LOC within individual subjects. In our trend judgment task, the pITG might be involved in extracting the orientation of the graphic*’*s principal axis, but it might also compute its length and width, respectively, along the primary and secondary axes, which, in the case of objects, are essential for both shape judgments and grasping tasks (Harris, 2024; McCloskey et al., 2006; Milner & Goodale, 1995).

### Possible Extension of the Cultural Recycling Hypothesis to Graphics

With the second research question, we asked whether the hypothesis of cultural recycling of cortical maps (Dehaene & Cohen, 2007; Dehaene, 2005), which has been primarily applied to literacy and numeracy, could be extended to graphics. The fact that the same pITG site was activated by judgments of object orientation and of the trend of a noisy scatterplot supports such a hypothesis by suggesting that the processes that detect object orientation are reused for graph trend judgments. In this respect, our finding is reminiscent of the observation that a subregion of ventral visual cortex involved in invariant object recognition gets recruited by the cultural activity of learning to read and becomes the visual word form area (Dehaene et al., 2015). In both cases, a novel cultural invention preempts an evolutionary older function. Naturally, the two domains differ in several other respects: Graphical perception requires attending to the visuospatial properties of objects, such as orientation and width, whereas reading requires attending to the identity of visual features, invariantly for size and orientation. It should therefore not be surprising that these two visual cultural activities are associated with very different cortical territories, although they both require cultural learning and recycling.

Compared with the vast literature on the brain mechanisms of reading, we readily acknowledge that the present experiment provides only initial and tentative support for the cultural recycling hypothesis of graphical perception. In the case of literacy, much evidence demonstrates that greater expertise in reading is associated with greater activation in the visual word form area (Dehaene et al., 2010, 2015), that expertise for reading in a specific script is reflected in this region (Szwed, Qiao, Jobert, Dehaene, & Cohen, 2014; Baker et al., 2007), and that dyslexics exhibit an abnormally reduced activation at this site (Feng et al., 2020). In the case of numeracy, we found that a nearby activation of the LOC/pITG in response to numbers and mathematical expressions was significantly enlarged in mathematicians compared with nonmathematicians (Amalric & Dehaene, 2016), suggesting that mathematical expertise can indeed affect the size of activation in this area. However, in the present case of graphicacy, we only found a positive correlation of activation with graphicacy expertise in the aIPS, but not in the pITG. Note that the present sample of participants did not vary much in terms of mathematical expertise (as STEM students, they were all highly proficient in mathematics). Studying a more heterogenous cohort of participants could also reveal differential activations of the pITG based on expertise, thus strengthening the cultural recycling hypothesis.

As mentioned in the Introduction section, the recycling hypothesis is also supported by behavioral work. When judging the trend of a noisy scatterplot, human adults do not minimize the vertical distance of the points to the linear trend, as they should if they were computing the ordinary least square regression; rather, they extract its principal axis (Ciccione & Dehaene, 2021), which is equivalent to the skeleton of physical objects (Ayzenberg et al., 2019; Bodily et al., 2018; Lowet, Firestone, & Scholl, 2018). This finding can be readily explained under the hypothesis that participants apply to graphics the same process of principal axis extraction that is used to determine the orientation of objects, even in situations where this procedure is suboptimal. Indeed, here as in previous studies, our noisy scatterplots were generated by adding noise only to the dependent variable *y*, whereas the independent variable *x* was fixed, that is, conditions under which the unbiased estimate is the ordinary least-squares slope, not the principal axis. The fact that participants extract the principal axis even though it is not the optimal estimator of the regression line seems a strange suboptimal quirk, but can be readily explained by the recycling hypothesis, according to which humans cannot help but apply a preexisting process of object orientation perception to the trend judgment task.

A third piece of evidence arises from behavioral judgments of correlation strength (rather than the slope, as we did). When humans are asked to rate the strength of the correlation in a scatterplot, their ratings grow non-linearly with the actual *r* (Rensink, 2017; Cleveland, Diaconis, & Mcgill, 1982). Furthermore, they are submitted to a strong illusion: The very same data points are perceived as showing a stronger *x*/*y* correlation when they are plotted over a smaller and more compact portion of the figure, which can be achieved by simply rescaling the axes (Cleveland et al., 1982). Again, both findings can be explained by the recycling hypothesis by assuming that participants treat the scatterplot as a single object and use its overall surface as a cue to correlation strength (with tightly grouped data suggesting a high correlation). Indeed, not only does a broadly dispersed data cloud suggest a weak correlation (Rensink, 2017), but the specific nonlinear relation between perceived and actual *r* approximately follows the equation $1-\sqrt{1-r^{2}}$, which is inversely related to the surface of the ellipsoid that encloses the dots $\left(\frac{\pi }{4}\sqrt{1-r^{2}}\right)$. Thus, both the nonlinearity and the bias for compactness in correlation estimation can be explained by a recycling of shape analysis.

An anonymous referee suggested that, for the construct of cultural recycling to have utility, it should be possible to point to cultural inventions that are not based on such recycling. However, we hold an opposite view (as already stated in Dehaene & Cohen, 2007; Dehaene, 2005). Because all cultural inventions, by definition, go beyond our evolutionary heritage, they must encroach on brain areas initially devoted to other functions, but plastic enough to adapt to a novel use. Furthermore, because cortex is not an isotropic learning machine, the neuronal recycling hypothesis predicts that there should be a limit to the interindividual and cross-cultural variations in how major inventions are acquired, which areas are involved, and how they operate. These predictions are largely upheld in the case of writing and arithmetic (Dehaene & Cohen, 2007), but remain to be studied in many other domains. The quest for the recycled brain areas sustaining them could be harder, as is the case for many sociocultural inventions (Parkinson & Wheatley, 2015), but finding which brain areas are recycled should prove very useful to understand the constraints that such recycling imposes. In the case of graphics perception, understanding them might elucidate: (1) the reasons why graphics are usually presented the way they are (to better accommodate the preexisting operations performed by the underlying areas); (2) the biases that humans exhibit in extracting information from some graphics (e.g., the Deming bias, as explained above); (3) why some graphics are easier to understand than others (possibly because they make a better use of existing neurocognitive processes); and (4) how graphics should be taught (by relying on preexisting intuitions, but also by overcoming them if needed). For all these reasons, we argue that the construct of neuronal recycling, precisely because of its generalizability to all cultural inventions, provides a useful theoretical framework for research in cognitive neuroscience (see also Nakai, Girard, Longo, Chesnokova, & Prado, 2023; Nordt et al., 2021; Lumaca, Kleber, Brattico, Vuust, & Baggio, 2019).

### Involvement of the Math-responsive Network during Trend Judgment

As an answer to our third research question, we also examined whether the brain activation evoked by graphics overlaps with that evoked by a classical arithmetic localizer (Pinel et al., 2004, 2007). A significant degree of overlap was found in the right anterior IPS (Figure 5). Previous research found that the IPS is recruited by a great variety of arithmetic and numerical tasks (Amalric & Dehaene, 2019; Dehaene, Piazza, Pinel, & Cohen, 2003), abstract high-level mathematical judgments (Amalric & Dehaene, 2016), and a variety of visuospatial tasks such as visuospatial working memory (Wang et al., 2019; Bray, Almas, Arnold, Iaria, & MacQueen, 2015), visuospatial transformations (Papadopoulos, Sforazzini, Egan, & Jamadar, 2018), topographical spatial maps (Silver & Kastner, 2009; Silver, Ress, & Heeger, 2005), numerotopic maps (Harvey, Klein, Petridou, & Dumoulin, 2013), and the mental number line (Berteletti, Man, & Booth, 2015). All these findings are in line with our graphic task, as scatterplots involve understanding how quantities are laid out in space. We note, however, that we observed a very anterior activation of the IPS, at the anterior margin of that observed during mental arithmetic and by most of the literature for mathematical tasks. One plausible reason is that most previous math tasks, including the present localizer, primarily consisted in arithmetic calculations: We chose this type of task only because it was shown to localize, in just 5 min of scanning, the math network (Pinel et al., 2007). It is likely that distinct aspects of mathematics relate to different sectors of the IPS. Future studies should examine, within single subjects, a greater variety of mathematical tasks, from graphical perception to number line, calculation, algebra, geometry, and logical reasoning, to clarify whether a systematic topological organization of the IPS can be found, and to find out which specific subdomains of mathematics induce brain activity that overlaps with our graphical perception task.

As visible from Figure 5A and C, we also found that trend judgments involved retrosplenial cortex. Such an involvement is far from absurd, as a similar activation is associated with different types of spatial cognition, including the spatial reference frames that allow us to orient and follow a route in the environment (Mitchell, Czajkowski, Zhang, Jeffery, & Nelson, 2018; Czajkowski et al., 2014; Galati, Pelle, Berthoz, & Committeri, 2010; Committeri et al., 2004). fMRI and intracranial recordings have shown retrosplenial cortex to be activated by spatial visual scenes as opposed to faces (Woolnough et al., 2020; Epstein & Baker, 2019), a contrast which bears a certain analogy to the present one. Although its function is currently illdefined, retrosplenial cortex is thought to be an integration region, “a critical interface between brain regions generating different forms of spatial mapping “ (Alexander & Nitz, 2015). As such, it makes sense that it would be activated by the very abstract sense of space elicited by a scatterplot on a computer screen. However, understanding its role is complicated by the fact that this area was generally deactivated by our stimuli and that its involvement with graphics was mostly due to a smaller deactivation during trend judgment than during proportion estimation (Figure 5D). Such a deactivation could, however, reflect the fact that this area is already strongly activated during the resting state, perhaps due to its constant computation of self-localization in spatial reference frames relative to our environment.

Taken together, these findings lend initial support for the kind of two-stage model presented in Figure 1. According to this view, the data in the scatterplot would first be treated as a single object, whose principal axis and its orientation would be extracted; the present data suggest that this may occur in pITG/LOC. Second, this information, as well as other properties such as the surface of the data, would be fed into higher areas of the math network, including the IPS, which would accumulate evidence about the mathematical properties of the graphic, such as the presence or absence of a relationship between *x*/*y* variables, its sign, strength, linear or nonlinear shape, and so forth. Such evidence accumulation, taking into account the level of noise, number of points, possible presence of outliers, and so forth, may occur in the aIPS. Although such an interpretation is clearly speculative, we note that a similar dissociation was proposed for the parallel involvement of posterior inferotemporal and intraparietal cortices in mental arithmetic, as evaluated by intracranial recordings (Pinheiro-Chagas, Daitch, Parvizi, & Dehaene, 2018). In the future, temporally resolved methods such as electroencephalography and magnetoencephalography or intracranial recordings will be needed to evaluate the dynamics of graphical perception.

Overall, independently from the veracity of the above model, the finding that the neural correlates of our trend judgment task were not restricted to occipital areas but spread to higher-level IPS and retrosplenial regions involved in higher spatial cognition and mathematics suggests that trend judgment might not be just a purely perceptual task and that the graphic may be perceived as a mathematical object. In agreement with this conclusion, in our sample of participants, we also observed a significant correlation in the aIPS between brain activation and the participants*’* individual graphicacy index. Previous larger-scale behavioral studies also showed that the performance in graphical perception tasks correlates tightly with math education and knowledge (Ciccione et al., 2022; Ludewig et al., 2020). However, this does not mean that, in the absence of math training, participants could not perform a simple trend judgment task. On the contrary, and as predicted by the neuronal recycling hypothesis, young children in preschool or first grade, unschooled adults from Namibia (Ciccione, Sablé-Meyer, et al., 2023), and even baboons (Ciccione et al., 2024) have been shown to be able to perform the graphic trend judgment task and to be influenced by the same *t*-value variable. We suggest that if graphical perception could be imaged in those populations, it would show a preexisting circuit for object orientation, that is, the first stage of our proposed two-stage process, but perhaps not the second stage, which involves understanding the mathematical meaning of the graphic, and depends on education to mathematics.

### Neural and Behavioral Dependence on the *t* Value of the Scatterplot

Lastly, our fourth research question was whether we could demonstrate the parametric dependence of behavioral judgments and brain activity on the *t* value of the scatterplot when performing trend judgments. At the behavioral level, we confirmed previous findings showing that, in their trend judgments, people rely on the *t* value of the graphic, which measures the statistical strength of the correlation (Ciccione, Sablé-Meyer, et al., 2023). Here, we extended these results by demonstrating that such reliance is independent of the visual hemifield in which the stimulus appears. Furthermore, at the neural level, we found that the *t* value of the graphic was a major determinant of brain activation, even when noise level was regressed out (Figure 6). In particular, the anterior IPS, which was significantly more activated for trend judgment than for proportion estimation, was also increasingly activated for decreasing levels of *t* values (i.e., for more difficult graphics). This finding further supports the involvement of the anterior IPS in graphical perception, showing that its activation is increased, presumably because of a greater duration of activation, when graphics are harder to judge. We can only speculate as to why pITG activity failed to be similarly modulated by *t* value in either direction. A tentative hypothesis, as we mentioned, is that pITG, being close to early visual regions of the occipital lobe, is just involved in the extraction of the global graphic layout and its orientation, which are only later converted into a trend-judgment decision (after being fed into the IPS).

## Conclusion

We close by stressing again that the trend judgment that we studied here is only a first step of the complex process of graphic understanding. In real-life situations, a reader would go through many successive stages when observing a graphic. For instance, after detecting the trend, she might use it to infer a relation between the variables, and therefore understand the meaning of the graphic and perhaps extrapolate future values based on the observed ones. All these processes are likely to involve brain areas over and above those identified here, and much more research will be needed to elucidate them. Meanwhile, the present work provides a first glimpse of the cortical areas involved in the initial stages of graphicacy.

## Supplementary Material

Supplementary materials

## Data Availability

First-level fMRI models, behavioral performance, and scripts for the analyses are available on the OSF network at https://osf.io/85fqj/. Supplemental Material can be accessed on this article*’*s homepage: https://10.1162/jocn.a.81.
