# Supplementary material for: The Neural Bases of Graphical Perception: A Novel Instance of Cultural Recycling?
Source: J Cogn Neurosci. Author manuscript; Available in PMC 2026 Jan 1. (PMC7618408; doi:10.1162/JOCN.a.81)
Supplement: Supplementary materials [file EMS210433-supplement-Supplementary_materials.docx]

**SUPPLEMENTAL ONLINE MATERIAL**

**The neural bases of graphical perception:**

**a novel instance of cultural recycling?**

# Authors

Lorenzo Ciccione^A, B, C^

Stanislas Dehaene^A, B^

^A^ Cognitive Neuroimaging Unit, CEA, INSERM, Université Paris-Saclay, NeuroSpin center, 91191 Gif/Yvette, France

^B^ Collège de France, Université Paris Sciences Lettres (PSL), 11 Place Marcelin Berthelot, 75005 Paris, France

^C^ DysCo Lab, Department of Psychology, Université Paris 8, 93526 Saint‑Denis, France.


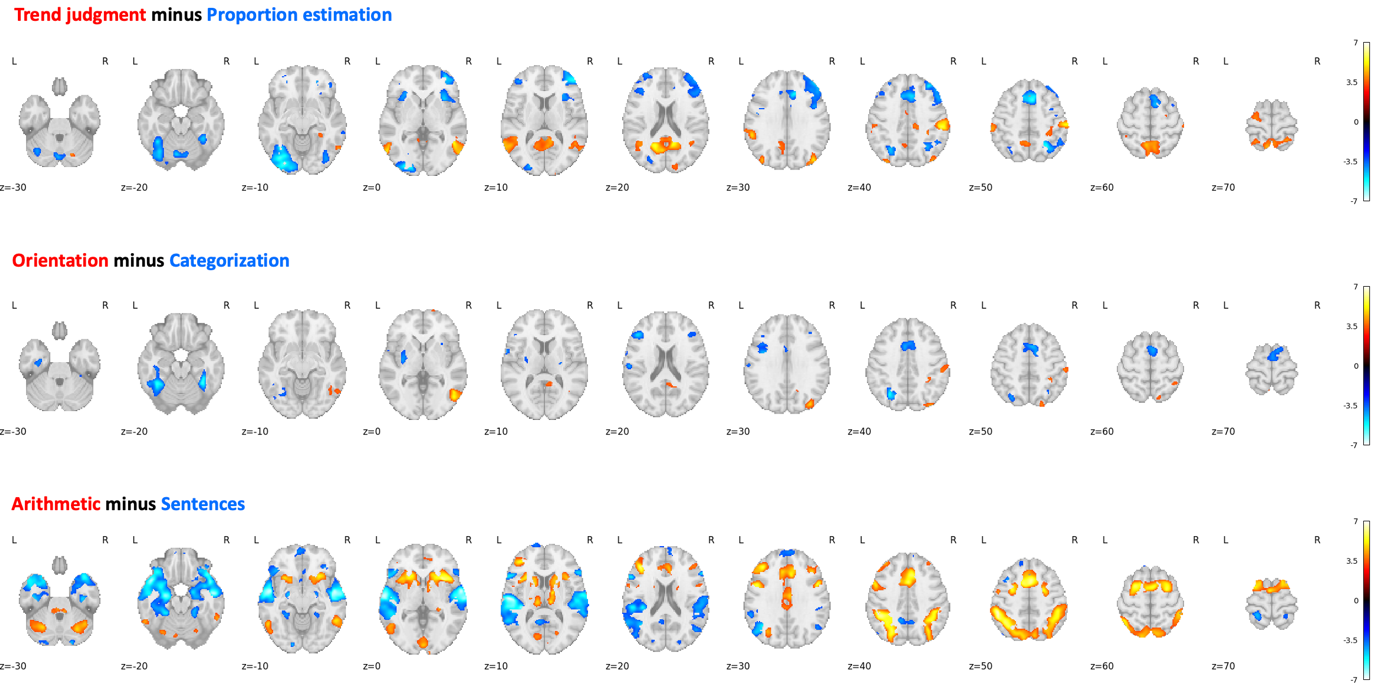


**Supplementary figure 1.** Full series of axial cuts for the main univariate contrasts (also shown in figure 3).


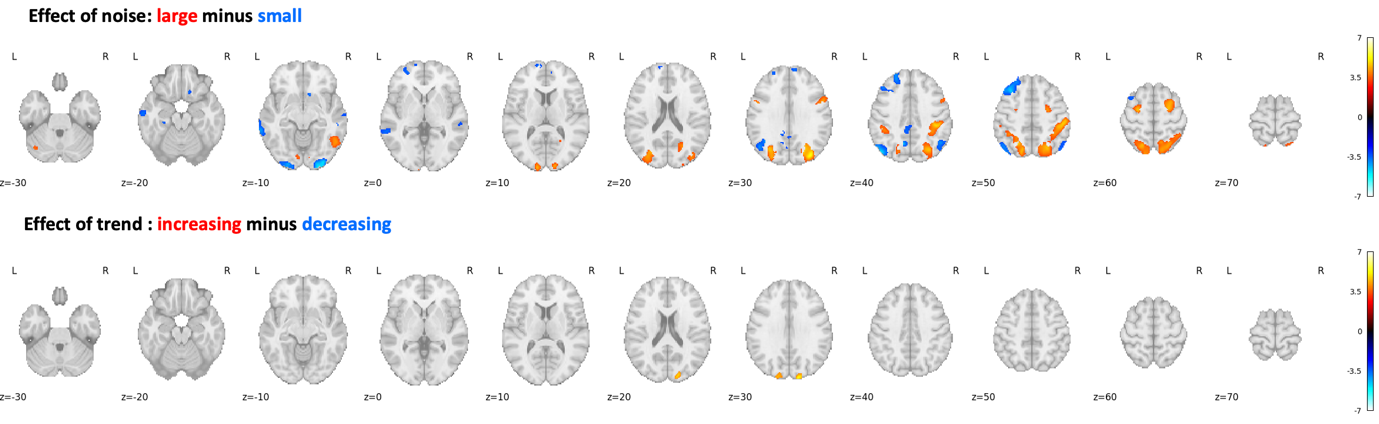


**Supplementary figure 2.** Full series of axial cuts showing the unmasked univariate contrasts that modulated the graphic trend judgement task in runs 2 and 4.


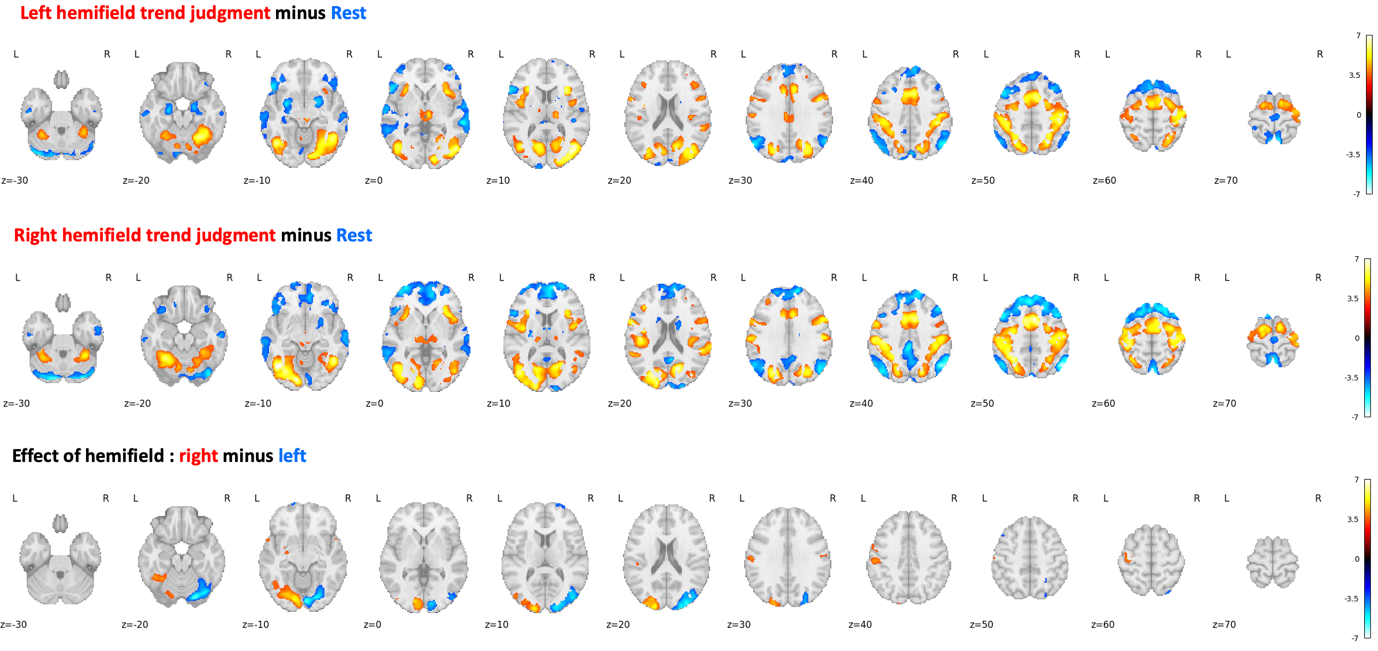


**Supplementary figure 3.** Full series of axial cuts showing the limited effect of hemifield on the graphic trend judgment task.


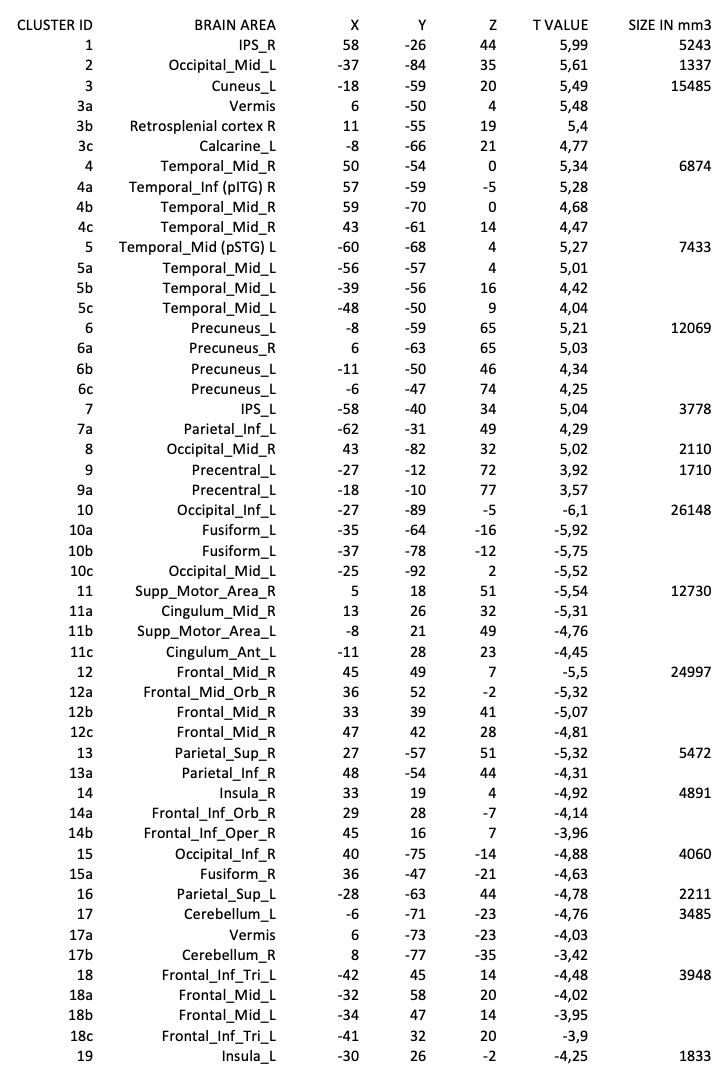


**Supplementary figure 4.** For the contrast *trend judgment > proportion detection*, each line gives the peak coordinates, volume and statistics of clusters (with p<.05 and size > 1 cm^3^). Letters indicate subpeaks within the same cluster. Coordinates are given in MNI space. Associated brain regions are extracted by the AAL (automated anatomical labelling) atlas.


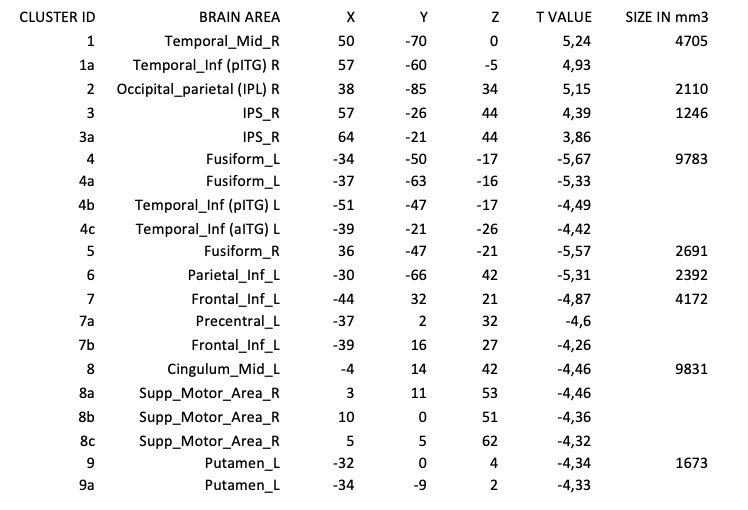


**Supplementary figure 5.** For the contrast *orientation judgment > identity judgment*, each line gives the peak coordinates, volume and statistics of clusters (with p<.05 and size > 1 cm^3^). Letters indicate subpeaks within the same cluster. Coordinates are given in MNI space. Associated brain regions are extracted by the AAL (automated anatomical labelling) atlas.


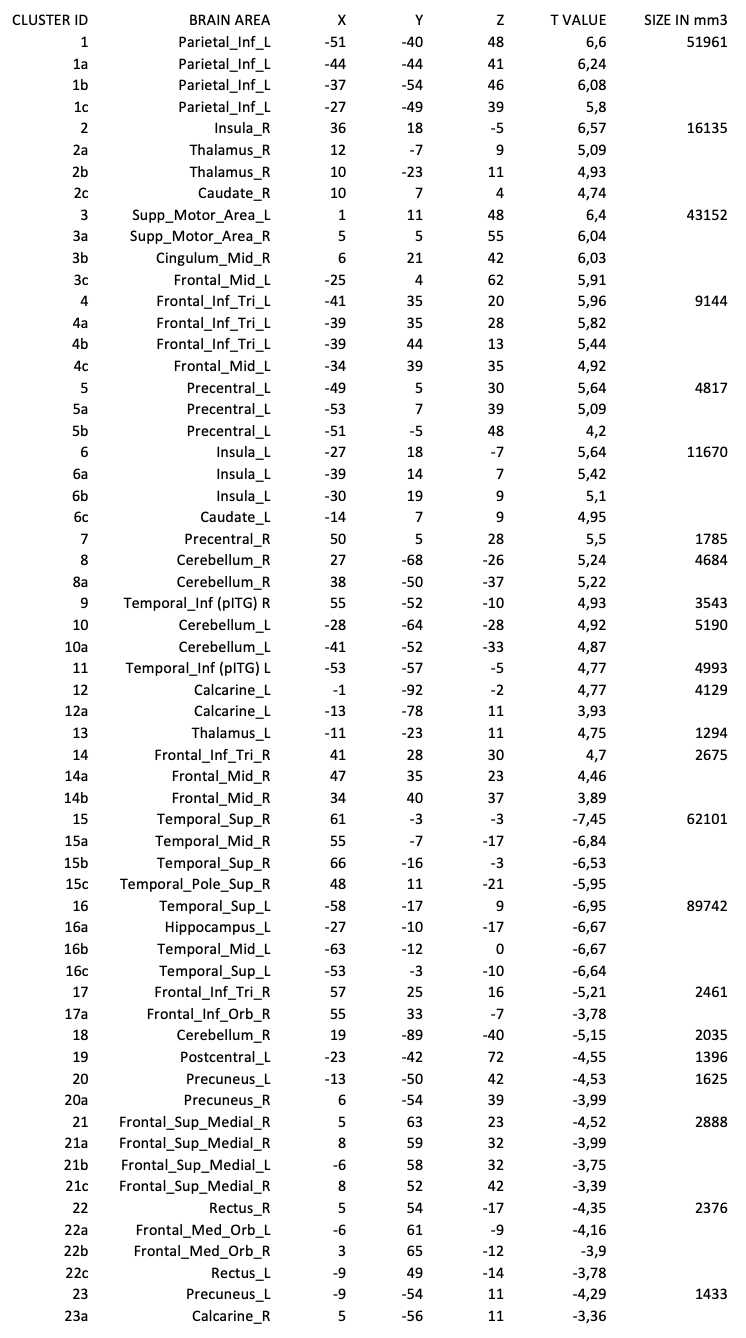


**Supplementary figure 6.** For the contrast *math > language*, each line gives the peak coordinates, volume and statistics of clusters (with p<.05 and size > 1 cm^3^). Letters indicate subpeaks within the same cluster. Coordinates are given in MNI space. Associated brain regions are extracted by the AAL (automated anatomical labelling) atlas.
